# Anthrax Epizootic in Wildlife, Bwabwata National Park, Namibia, 2017

**DOI:** 10.3201/eid2505.180867

**Published:** 2019-05

**Authors:** Caitlin M. Cossaboom, Siegfried Khaiseb, Bernard Haufiku, Puumue Katjiuanjo, Apollinaris Kannyinga, Kaiser Mbai, Thompson Shuro, Jonas Hausiku, Annety Likando, Rebekka Shikesho, Kofi Nyarko, Leigh Ann Miller, Simon Agolory, Antonio R. Vieira, Johanna S. Salzer, William A. Bower, Lindsay Campbell, Cari B. Kolton, Chung Marston, Joy Gary, Brigid C. Bollweg, Sherif R. Zaki, Alex Hoffmaster, Henry Walke

**Affiliations:** Centers for Disease Control and Prevention, Atlanta, Georgia, USA (C.M. Cossaboom, A.R. Vieira, J.S. Salzer, W.A. Bower, C.B. Kolton, C. Marston, J. Gary, B.C. Bollweg, S.R. Zaki, A. Hoffmaster, H. Walke);; Republic of Namibia Ministry of Agriculture, Water, and Forestry, Windhoek, Namibia (S. Khaiseb, K. Mbai, T. Shuro);; Republic of Namibia Ministry of Health and Social Services, Windhoek (B. Haufiku, P. Katjiuanjo, A. Likando, R. Shikesho, K. Nyarko);; Republic of Namibia Ministry of Environment and Tourism, Windhoek (A. Kannyinga, J. Hausiku);; Centers for Disease Control and Prevention, Windhoek (L.A. Miller, S. Agolory); University of Florida, Vero Beach, Florida, USA (L. Campbell)

**Keywords:** anthrax, Bacillus anthracis, wildlife, zoonoses, Namibia, Africa, control, epizootic, bacteria

## Abstract

In late September 2017, Bwabwata National Park in Namibia experienced a sudden die-off of hippopotamuses and Cape buffalo. A multiorganizational response was initiated, involving several ministries within Namibia and the US Centers for Disease Control and Prevention. Rapid interventions resulted in zero human or livestock cases associated with this epizootic.

Anthrax, caused by *Bacillus anthracis*, is a naturally occurring zoonotic disease of veterinary and public health importance. Anthrax has been reported in wildlife and domestic animals worldwide and can spill over to humans (*1**,**2*). Anthrax epizootics in hippopotamuses have been documented in several countries of southern Africa, including Zambia, Zimbabwe, and South Africa (*3**–**5*). Anthrax is also well documented in wildlife in Etosha National Park, Namibia (*6*). Human infections related to wildlife anthrax typically result from consumption of meat from infected carcasses, causing ingestion anthrax, or direct contact, causing cutaneous anthrax (*5*). Anthrax epizootics in southern Africa are often associated with dry seasons, which typically occur during May–October (*2**,**7*).

A massive dieoff of hippopotamuses and Cape buffalo began in late September 2017 along the Kavango River in Bwabwata National Park (BNP), within the Kavango East region of northeastern Namibia. We report on the multiorganizational response that addressed this event.

## The Study

On September 25, 2017, Namibia’s Ministry of Environment and Tourism (MET) learned of 2 hippopotamus carcasses found on the western side of BNP. During unrelated aerial surveillance in the area on October 1, MET observed 10 hippopotamus carcasses and on October 7 observed an additional 107 hippopotamus and 20 Cape buffalo carcasses. MET then notified the Ministry of Agriculture, Water, and Forestry and the Ministry of Health and Social Services (MOHSS), which together established a joint action plan. The presumptive diagnosis was anthrax based on clinical and microscopic evidence; however, culture confirmation was not initially possible because of sample collection and laboratory challenges. The US Centers for Disease Control and Prevention (CDC) was consulted for assistance with the investigation.

**Figure 1:**
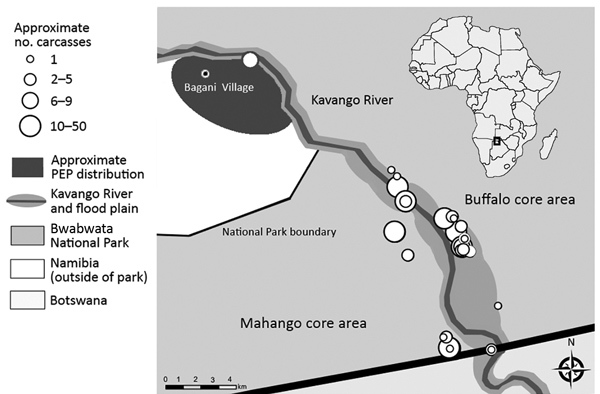
Anthrax investigation points of interest within and directly adjacent to Bwabwata National Park, Namibia, 2017. Inset shows location of park in Africa.

To address possible human exposure, MOHSS developed a questionnaire and administered it beginning on October 12 to identify persons living in communities adjacent to BNP who were exposed to the carcasses (Figure 1). In total, 1,050 persons were identified as having contact with or consuming meat from the carcasses and were immediately provided with postexposure prophylaxis (PEP) in the form of 2 weeks of ciprofloxacin and symptom monitoring (*8*). MOHSS returned 2 weeks later to assess PEP adherence among exposed community members and reported successful PEP adherence and no severe adverse events associated with taking PEP. Field workers performing carcass disposal continued PEP use until 2 weeks after activities concluded. MOHSS led community education to raise awareness of the ongoing outbreak, communicate the importance of not having contact with or consuming meat from animals found dead, and urge persons to seek healthcare if exposed or symptomatic. No human anthrax cases were associated with this outbreak.

CDC developed a protocol to collect specimens from affected wildlife carcasses. A field team collected several paired samples from 7 carcasses: ear and eyelid tissue biopsy samples and swab specimens of the nasal cavity, rectum, and other pooled blood when available. We recorded geographic coordinates by using a Garmin Montana 650 GPS unit at carcass locations for eventual geospatial analysis (Figure 1).

To test samples, we used the InBios Active Anthrax Detect (AAD) Rapid Test (InBios, http://www.inbios.com), a lateral flow assay that detects capsular polypeptide of *B. anthracis*. This novel assay was developed as a point-of-care diagnostic aid for human inhalation anthrax and is available for investigational or research use only (*9*). A laboratory evaluation conducted on animal tissues by CDC before deployment indicated that the test had 98% specificity and 95% sensitivity (C.B. Kolton, unpub. data). Use of the AAD Rapid Test under field conditions is beneficial because the test requires only a small sample volume, provides results within 15 minutes, and does not require refrigeration.

We performed the AAD Rapid Test in the field on tissue and swab samples collected from wildlife carcasses, following standard protocol provided by InBios (S. Raychaudhuri, InBios, pers. comm., 2016 May 10). We suspended tissue samples in 600 µL of sterile phosphate-buffered saline (PBS) and vortexed the suspension for 10 seconds. After pipetting repeatedly, we applied 10 µL to the AAD Rapid Test cassette. For swabbed exudate samples, we transferred 10 µL of fluid to the cassette without PBS.

We subsequently confirmed *B. anthracis* infection by using culture, real-time reverse transcription PCR (rRT-PCR), and immunohistochemistry (IHC). We processed tissue and swab samples and inoculated them into sheep blood agar or heart infusion broth, then incubated at 37°C for 24 h. We performed DNA extractions on specimens by using the QIAGEN Blood Mini Kit (https://www.qiagen.com) and tested the resulting DNA by using the Laboratory Response Network’s rRT-PCR for *B. anthracis* (*10*). We processed formalin-fixed tissue samples from 3 Cape buffalo and 3 hippopotamuses, embedded them in paraffin, and stained them with hematoxylin and eosin, Lillie-Twort Gram stain, and Warthin-Starry silver stain. 

**Figure 2:**
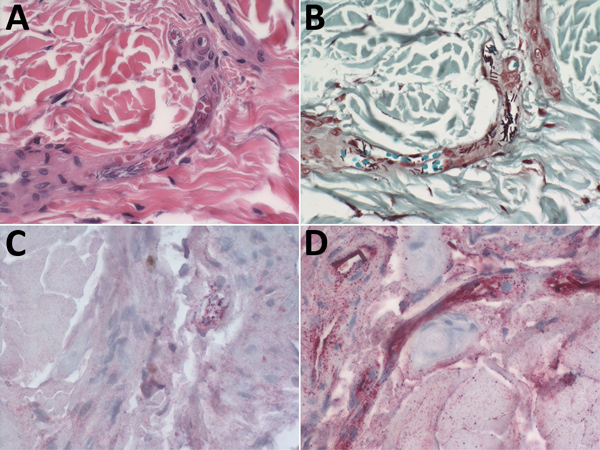
Photomicrographs showing hematoxylin and eosin stain and immunohistochemical findings, using assays targeting the cell wall and capsule of *Bacillus anthracis*, in ear-punch biopsy specimens from a hippopotamus infected with *B. anthracis*, Bwabwata National Park, Namibia, 2017. A) Hematoxylin and eosin stain showing large bacilli evident in vessel lumen. Original magnification × 40. B) Gram stain showing gram-variable rods evident in vessels. Original magnification × 40. C) Immunohistochemical stain of *B. anthracis* cell wall showing antigen evident in vessels (red). Original magnification × 40. D) Immunohistochemical stain of *B. anthracis* capsule showing bacilli evident in vessels (red), and bacterial antigen. Original magnification × 63.

The vessels within the dermis of the ear biopsy in 1 Cape buffalo and ear and eyelid biopsies of 3 hippopotamuses contained large, gram-variable bacilli. We performed IHC assays using mouse monoclonal antibodies targeting the *B. anthracis* cell wall and capsule by using an immunoalkaline phosphatase polymer system, as previously described (*11**,**12*), and highlighted bacterial antigen and full bacilli within the vessels of 2 buffalo and all 3 hippopotamuses (Table; Figure 2). All 6 anthrax-suspected carcasses tested positive by AAD Rapid Test and were confirmed positive for *B. anthracis* by culture, rRT-PCR, and/or IHC. A carcass of a buffalo that died from a vehicle collision was included as a negative control and tested negative by all assays (Table).

**Table:** Summary of laboratory diagnostic testing results, by carcass sampled, after an anthrax epizootic in wildlife, Bwabwata National Park, Namibia, 2017.

Carcass ID	Species	AAD Rapid Test	Culture	LRN rRT-PCR	Immunohistochemistry	
					Cell wall	Capsule
001	Cape buffalo	+	+	NA	–	+
002	Cape buffalo†	–	–	NA	–	–
005	Cape buffalo	+	+	NA	+	+
007	Hippopotamus	+	+	+	+	+
008	Hippopotamus	+	–	+	+	+
009	Hippopotamus	+	+	+	+	+
012	Hippopotamus	+	+	+	NA	NA

*AAD Rapid Test, InBios Active Anthrax Detect Rapid Test (InBios, http://www.inbios.com); LRN rRT-PCR, Laboratory Response Network real-time reverse transcription PCR; NA, not available; +, positive; –, negative. †This Cape buffalo carcass served as a negative control. The animal died as a result of a vehicle collision, and anthrax infection was not suspected as cause of death.

Livestock vaccination is an effective means to prevent anthrax infection in domestic animals and subsequent transmission to humans (*13*) and is required annually in Namibia. The Ministry of Agriculture, Water, and Forestry secured ≈10,000 doses of livestock anthrax vaccine to prevent spillover of anthrax from wildlife into susceptible domestic animals and will enforce future annual vaccination in the affected area. MET organized vaccination of wildlife. No cases of anthrax in livestock were associated with this outbreak. 

Incineration is the recommended disposal method in cases where reducing the carcass to ashes is possible to enable complete destruction of any viable spores (*8*). However, because of the large size and number of carcasses, burial was an alternative method of disposal. We recommended limiting the distance that carcasses were moved for burial to decrease dissemination of spores and ensuring a depth of >2 m was reached to prevent scavenger disruption to carcasses (*1*). Spraying the carcass and burial site with 10% formaldehyde minimized external contamination (*1**,**8*).

By early December, the wildlife deaths subsided, and 155 hippopotamus and 86 Cape buffalo carcasses had been disposed of in the Mahango and Buffalo core areas of BNP (Figure 1). MET conducted an aerial survey in the same areas in September 2017 that recorded 588 hippopotamuses and 2,216 Cape buffalo; roughly 26.4% and 3.8% of each population were affected, respectively (*14*).

## Conclusions

Our investigation highlights a successful public health outcome with zero human or livestock cases after an anthrax outbreak in wildlife in BNP. We demonstrated the successful use of the AAD Rapid Test for presumptive diagnosis of anthrax in wildlife under field conditions and the use of culture and IHC for confirmation of *B. anthracis* in hippopotamuses and Cape buffalo. The AAD Rapid Test has the potential to improve the ability of low-resource countries to quickly diagnose and effectively manage anthrax epizootics, thus reducing the risk for transmission to humans.

Swift response in organizing PEP dissemination, community education, livestock vaccination, and carcass disposal contributed to the prevention of anthrax transmission to humans and livestock. Our investigation emphasizes the importance of a multiagency coordinated response for zoonotic disease outbreaks and continued efforts to raise awareness of the risks of consuming meat from animal carcasses in anthrax-endemic areas.
